# An Integrated Model of Emotional Problems, Beta Power of Electroencephalography, and Low Frequency of Heart Rate Variability after Childhood Trauma in a Non-Clinical Sample: A Path Analysis Study

**DOI:** 10.3389/fpsyt.2017.00314

**Published:** 2018-01-22

**Authors:** Min Jin Jin, Ji Sun Kim, Sungkean Kim, Myoung Ho Hyun, Seung-Hwan Lee

**Affiliations:** ^1^Clinical Emotion and Cognition Research Laboratory, Inje University, Goyang, South Korea; ^2^Department of Psychology, Chung-Ang University, Seoul, South Korea; ^3^Department of Psychiatry, Soonchunhyang University Cheonan Hospital, Cheonan, South Korea; ^4^Department of Biomedical Engineering, Hanyang University, Seoul, South Korea; ^5^Department of Psychiatry, Inje University, Ilsan-Paik Hospital, Goyang, South Korea

**Keywords:** childhood trauma, heart rate variability, low-frequency power, electroencephalography, beta, affective lability

## Abstract

Childhood trauma is known to be related to emotional problems, quantitative electroencephalography (EEG) indices, and heart rate variability (HRV) indices in adulthood, whereas directions among these factors have not been reported yet. This study aimed to evaluate pathway models in young and healthy adults: (1) one with physiological factors first and emotional problems later in adulthood as results of childhood trauma and (2) one with emotional problems first and physiological factors later. A total of 103 non-clinical volunteers were included. Self-reported psychological scales, including the Childhood Trauma Questionnaire (CTQ), State–Trait Anxiety Inventory, Beck Depression Inventory, and Affective Lability Scale were administered. For physiological evaluation, EEG record was performed during resting eyes closed condition in addition to the resting-state HRV, and the quantitative power analyses of eight EEG bands and three HRV components were calculated in the frequency domain. After a normality test, Pearson’s correlation analysis to make path models and path analyses to examine them were conducted. The CTQ score was significantly correlated with depression, state and trait anxiety, affective lability, and HRV low-frequency (LF) power. LF power was associated with beta2 (18–22 Hz) power that was related to affective lability. Affective lability was associated with state anxiety, trait anxiety, and depression. Based on the correlation and the hypothesis, two models were composed: a model with pathways from CTQ score to affective lability, and a model with pathways from CTQ score to LF power. The second model showed significantly better fit than the first model (AIC_model1_ = 63.403 > AIC_model2_ = 46.003), which revealed that child trauma could affect emotion, and then physiology. The specific directions of relationships among emotions, the EEG, and HRV in adulthood after childhood trauma was discussed.

## Introduction

Childhood trauma is regarded as a risk factor in the development of various affective disorders in childhood as well as in adulthood (1). Children with early trauma have an increased risk for the development of depression and anxiety disorders (2–4), and they experience greater mood lability than those that did not experience significant childhood trauma (5, 6).

Childhood trauma is associated with changes in brain function (7). Research using functional magnetic resonance imaging has discovered hyperactive amygdala activation (8), greater activation in the prefrontal cortex and basal ganglia (9), and reduced functional activity of the cerebellar vermis (10) in adults with childhood maltreatment. Research using electroencephalography (EEG) has found that the score of childhood trauma was significantly correlated with left frontal alpha power and with left parietal theta, alpha, and beta when EEG was tested at resting eyes closed state (11). A recent study also found that a group with high childhood trauma score showed significantly increased delta, beta and gamma power, and significantly decreased low alpha power in addition to significantly decreased anxiety and depression levels compared to a group with low childhood trauma scores (12).

Childhood trauma is also associated with biological changes, including heart rate variability (HRV), which is widely used to explain symptoms related to children’s stress status (13) and posttraumatic stress disorder (PTSD) (14). HRV offers a reliable measure of cardiovascular autonomic responses, and the frequency domains of HRV consist of high-frequency (HF), low-frequency (LF), and very-low-frequency (VLF) components (15). HF represents exclusively parasympathetic activity (16), and VLF reflects neuroendocrine and thermoregulatory influences on the heart (17), renin–angiotensin–aldosterone modulation (18), and vagal withdrawal (19). LF is proposed to reflect a complex mix of sympathetic, parasympathetic, and other unidentified factors (20). Since HRV is known to be associated with various regulations and to react to environmental changes (21–23), it could be related to traumatic environmental experiences. Adverse childhood experiences have a negative impact on systolic blood pressure representing sympathetic cardiovascular activity (24).

The heart constantly interacts with the brain (23). One study found that the LF power of HRV was negatively correlated with delta, sigma, and beta bands (25), while HF power was positively related to the EEG delta band in sleep apnea patients (25, 26). Studies of patients with PTSD have found significantly lower LF and HF power compared to healthy subjects (27), as well as increased theta and beta bands (28) and decreased alpha band (29). PTSD patients showed significantly increased theta activity in parietal lobes and frontal lobes (30, 31), as well as increased alpha connectivity (31) than control groups. Another study also found that PTSD patients showed higher peak alpha and lower HF than healthy controls (32). This connection between the heart and the brain is known to be related to the experience of emotions (33–35).

Indices of resting qEEG have been related to emotional problems, including affective lability, anxiety, and depression. Alpha waves are typical for an alert, but relaxed mental state, whereas beta activity is connected with an alert state of mind (36). Alpha rhythm was found to be correlated with emotional instability, which includes affective lability (37). Individuals with high anxiety have shown smaller alpha rhythms, greater delta rhythms, and greater HF beta rhythms than individuals with low anxiety (37), while depressed individuals have shown elevated alpha and beta frequencies compared to the controls (38).

As stated above, childhood trauma is known to be related to not only emotional problems but also qEEG indices (11, 12) and HRV indices (24) in adulthood. In addition, these factors could be related to one another simultaneously (39, 40). However, the exact directions between these factors have not been reported yet, and some pathway models involved with childhood trauma did not fully cover these various factors (12). In particular, some argue that the heart and brain change in response to an emotional stimulus to produce the experience of emotions (34, 35), while others claim that emotions change the signals that brain sends to the heart (41). Although childhood trauma, as stated above, is related to emotional problems and physiological changes, the sequence of what happens after childhood trauma is unclear yet. Therefore, this study aimed to depict two models that explain pathways after childhood trauma including emotional problems, such as depression, anxiety, affective lability, and physiological parameters, such as qEEG and HRV indices, and to reveal whether child trauma could first affect emotions or physiology. We hypothesized two extensive models: (1) one with physiological factors first and emotional problems later in adulthood as results of childhood trauma and (2) one with emotional problems first and physiological factors later.

## Materials and Methods

### Participants

The participants were 107 non-clinical volunteers without any history of psychoactive medication who volunteered after seeing advertisements for this study. Through an initial interview by a psychiatrist, individuals who did not have any formal diagnosis of neurological or other mental diseases including posttraumatic stress disorder both in the past and at the time of the study were included in the study. In addition, all participants had normal or corrected to normal vision and hearing ability. People who were pregnant, had substance use history, or had high suicidal risk were excluded from the study. Three individuals were excluded due to insufficient epochs for the qEEG analysis, and one subject was excluded due to careless and dismissed responses. The number of the final sample were 103, and this was larger than the standard minimum sample size of path analyses, which is 100 (42). The final sample consisted of 64 (61.5%) women and 39 (37.9%) men with a mean age of 28.18 years (SD = 6.18). The mean years of education was 14.74 (SD = 1.65). All procedures followed were in accordance with the ethical standards of the Institutional Review Board (2015-07-026-001) and with the Declaration of Helsinki. Informed consent was obtained from all participants for being included in the study.

### Psychological Measures

The Korean-validated version of the *Childhood Trauma Questionnaire* (CTQ) was used to assess childhood trauma. The CTQ consists of five trauma subscales, including emotional abuse, physical abuse, sexual abuse, emotional neglect, and physical neglect, and also additional minimization/denial subscales for detecting extreme response bias (43). It consists of 28 items and is assessed on a 5-point Likert scale, and the range of possible scores is 28–140. Scores below 31 suggest that individuals experienced very little trauma or no trauma in childhood, scores between 41 and 51 suggest that they experienced mild to moderate trauma, scores between 56 and 68 suggest that they experienced moderate to severe trauma, and scores above 73 suggest that they experienced severe to extreme trauma. It is a well-proven scale for evaluating a history of childhood trauma, even though it is assessed retrospectively. In this study, Cronbach’s α for internal consistency reliability was 0.90.

The Korean-validated version of the *State–Trait Anxiety Inventory* was used to evaluate state anxiety, which is more temporary, and trait anxiety, which is more general and long-lasting quality (44). It has 40 items assessed on a 4-point Likert scale, and the range of possible scores is 20–80 for trait anxiety and state anxiety, respectively. Scores of up to 30 suggest that individuals have no anxiety to low anxiety, and scores above 30 suggest that they have moderate to high anxiety, based on previously reported cutoff scores (45). In this study, Cronbach’s α for internal consistency reliability was 0.91 for state anxiety and 0.94 for trait anxiety.

The Korean-validated version of the *Beck Depression Inventory-I* was employed to measure depressive symptoms (46). It is composed of 21 items assessed on a 4-point Likert scale, and the range of possible scores is 0–63. A total score of 0–9 indicates that a person is not depressed, 10–15 indicates mild depression, 16–23 indicates moderate depression, and 24–63 signifies indicates severe depression (47). In this study, Cronbach’s α for internal consistency reliability was 0.90.

The Korean short form of the *Affective Lability Scale* (ALS) was utilized to estimate the instability of mood, which refers to rapid shifting between different emotional states (48). It consists of three subscales, namely anxiety/depression, depression/elation, and anger, and includes 18 items assessed on a 4-point Likert scale. The range of possible scores is 0–54. Cronbach’s α for internal consistency reliability in this study was 0.94.

### EEG Acquisition and qEEG Analysis

Participants were asked not to consume caffeine or alcoholic beverages and not to do immoderate exercise the day before the experiment. The participants came to the laboratory at 10:00 a.m. or 2:00 p.m. First, they completed the questionnaires/scales for 20 min. Second, resting-state EEG was recorded with their eyes open and eyes closed for 3 min, respectively. Finally, ECG signal was measured for 5 min. The whole procedure took about 1 h to complete.

The participants were seated in a comfortable chair in a sound-attenuated room. Resting-state EEG was recorded with subjects’ eyes open and eyes closed for 3 min each. The EEG signal was acquired by a NeuroScan SynAmps amplifier (Compumedics USA, E1 Paso, TX, USA) with 62 Ag–AgCl electrodes (FP1, FPZ, FP2, AF3, AF4, F7, F5, F3, F1, FZ, F2, F4, F6, F8, FT7, FC5, FC3, FC1, FCZ, FC2, FC4, FC6, FT8, T7, C5, C3, C1, CZ, C2, C4, C6, T8, TP7, CP5, CP3, CP1, CPZ, CP2, CP4, CP6, TP8, P7, P5, P3, P1, PZ, P2, P4, P6, P8, PO7, PO5, PO3, POZ, PO4, PO6, PO8, CB1, O1, OZ, O2, and CB2) mounted on a Quik Cap using an extended 10–20 placement scheme. The ground electrode was located on the forehead and the reference electrode was attached to both mastoids. The vertical electrooculogram (EOG) was positioned above and below the left eye and the horizontal EOG was recorded at the outer canthus of each eye. The impedance was kept below 5 kΩ. All data were processed with a 0.1–100 Hz band pass filter at a sampling rate of 1000 Hz, with 60 Hz noise removed using a notch filter.

**Figure 1:**
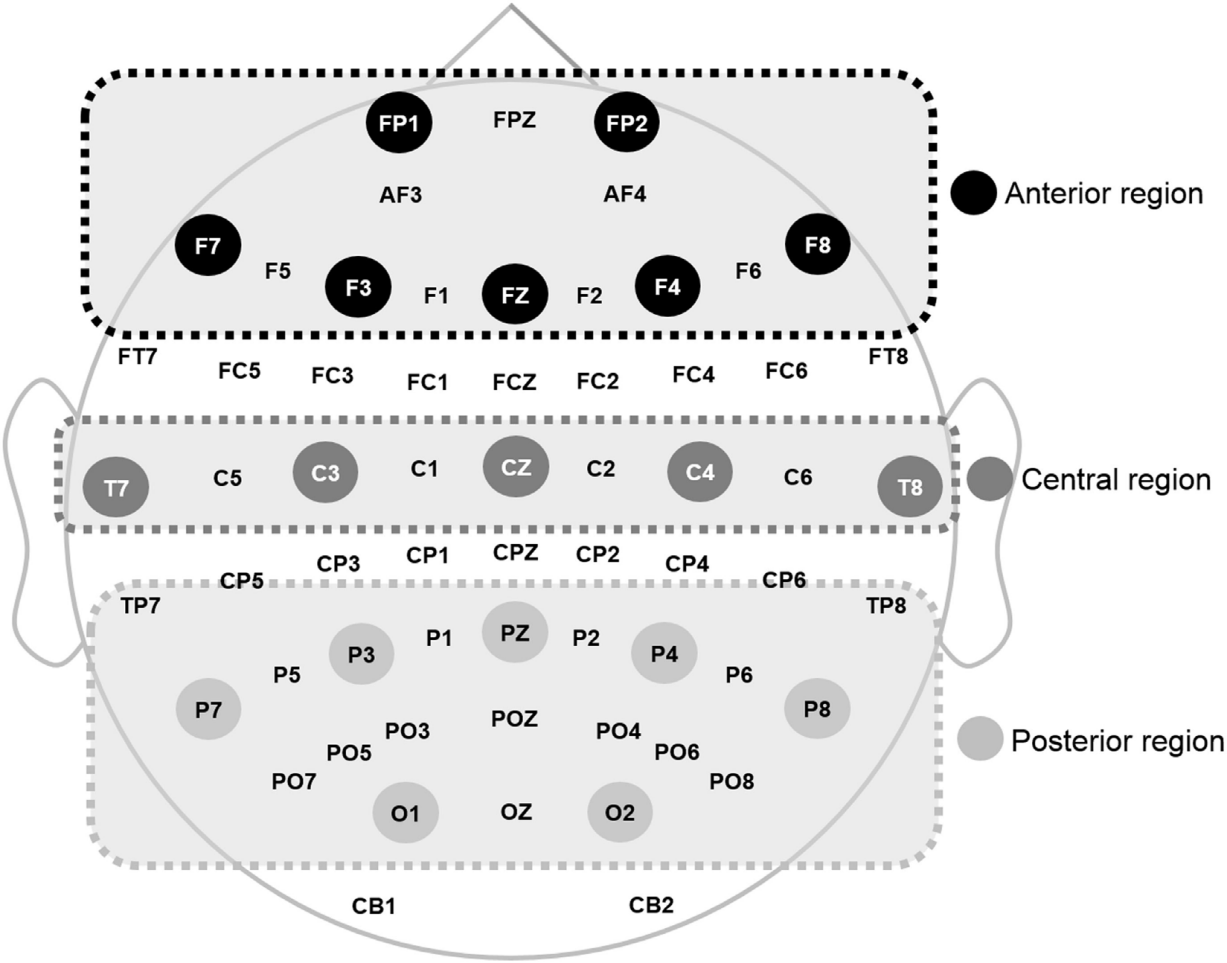
Electroencephalography electrode used and the regions 1 where power was estimated.

The recorded EEG data were preprocessed using CURRY 7 and MATLAB 2014a (MathWorks Inc., Natick, MA, USA). Eye movements were visually screened and eliminated by a trained person with no prior information regarding data origin. In this study, the resting EEG data with eyes closed were analyzed. EEG data were divided into epochs with a length of ~2 s (2,048 points) and the epochs with signals exceeding ±100 µV on any channel were excluded from further analysis. A total of 30 epochs (~60 s) were prepared for each participant. A fast Fourier transformation was performed for division into eight frequency bands: delta (1–4 Hz), theta (4–8 Hz), alpha1 (8–10 Hz), alpha2 (10–12 Hz), beta1 (12–18 Hz), beta2 (18–22 Hz), beta3 (22–30 Hz), and gamma (30–50 Hz) (49). Then, the relative power of each electrode was calculated by dividing each band power by the total power of the electrode. Relative power was averaged into three regions: anterior (FP1, F3, F7, Fz, FP2, F4, and F8), middle (T7, C3, Cz, T8, and C4), and posterior (P7, P3, O1, Pz, P8, P4, and O2), as shown in Figure 1, based on previous qEEG research (50). Relative global band powers were calculated over 62 electrodes and then averaged (51).

### Heart Rate Variability

Electrocardiography (ECG) signal was measured for 5 min using QECG-3: LXC3203 (Laxtha, Daejeon, Korea) and the sampling rate was 256 Hz. Standard three-limb leads (lead I, lead II, and lead III) were applied. The ECG electrode sensors were attached to the participants and they were asked to breathe normally with their eyes open and to sit quietly without moving. The recorded ECG data were analyzed using TeleScan (ver. 2.0, Laxtha) in the frequency domains according to task force recommendations (22). A high-pass filter with a cutoff frequency of 2 Hz was applied to the recorded ECG data for artifact rejection. RR-interval variability was extracted using the filtered ECG data. The power spectrum of the HRV signal was calculated using Fourier transformation, and power (area under the curve related to each component) was calculated for three components: VLF power (<0.04 Hz), LF power (0.04–0.15 Hz), and HF power (0.15–0.4 Hz), expressed in units of s^2^/Hz.

### Data Analysis

Normality was tested for each variable before further analysis. Skewness over 2.0 and kurtosis over 7.0 were considered to reflect a moderately non-normal distribution (52). Pearson’s correlation analysis was performed with the total scores of CTQ, psychological measures, qEEG indices, and HRV indices by generating 1,000 bootstrapped samples for multiple comparisons (53). All statistical analyses were conducted using SPSS 21.0. Based on the correlation results, two models were hypothesized: (1) one with physiological factors first and emotional problems later in adulthood as results of childhood trauma and (2) one with emotional problems first and physiological factors later. Path analyses to determine a better-fit model were performed with the maximum likelihood estimator using AMOS 21.0. To evaluate the fitness of each model, chi-square test (χ^2^), comparative fit index (CFI), Tucker–Lewis index (TLI), root mean square error of approximation (RMSEA), and standardized root mean squared residual (SRMR) indices were used, and the following cutoff points were adopted: CFI, TLI >0.95; SRMR <0.08; and RMSEA <0.06 (54). For model comparison, a comparison with the Akaike Information Criterion (AIC) index was performed. The significance level was set at *p* < 0.05 (two-tailed).

## Results

### Descriptive Statistics and Correlation

The mean score for CTQ was 42.29, which means individuals had experienced mild to moderate childhood trauma. Although participants were non-clinical volunteers, 47.5% showed that they had experienced at least mild to moderate childhood trauma, and 9.9% had experienced at least moderate to severe childhood trauma. The mean scores on the five subscales of CTQ were as follows: 9.05 (SD = 6.25) for emotional neglect, 7.28 (SD = 2.7) for physical abuse, 5.81 (SD = 1.92) for sexual abuse, 6.35 (SD = 2.53) for emotional abuse, and 6.17 (SD = 1.97) for physical neglect, and 7.64 (SD = 2.86) for minimization/denial. The minimization/denial subscales of CTQ were calculated to verify the possibility of false positive, and all participants showed scores over 3, which means that the bias was not substantial (43). While the total CTQ scores were normally distributed, the subscales such as sexual abuse, emotional abuse, and physical neglect were not since the participants were non-clinical. Therefore, the total CTQ score was utilized for further analyses.

All variables in our results, including CTQ scores, were within the range of normal distribution except for posterior beta3 (skewness was 2.76), posterior gamma (skewness was 2.95, and kurtosis was 7.26), and global gamma (skewness was 2.4). These three variables that were not normally distributed were log-transformed to be normally distributed for further parametric analyses.

Correlation analysis was executed in order to establish hypothesized models for further path analyses. Correlation coefficients, mean, and SD of key variables are presented in Table 1. Correlation analysis revealed that the total score for childhood trauma was significantly correlated with depression (*r* = 0.470, *p* < 0.001), state (*r* = 0.349, *p* < 0.001) and trait (*r* = 0.471, *p* < 0.001) anxiety, affective lability (*r* = 0.301, *p* = 0.002), and the LF power of HRV (*r* = −0.227, *p* = 0.021). Interestingly, while the childhood trauma score was not significantly correlated with any frequency band of qEEG, LF power was significantly correlated with some beta qEEG bands, such as anterior beta1 (*r* = −0.231, *p* = 0.019), anterior beta2 (*r* = −0.260, *p* = 0.008), anterior beta3 (*r* = −0.201, *p* =0.041), middle beta1 (*r* = −0.245, *p* =0.013), middle beta2 (*r* = −0.232, *p* = 0.018), middle beta3 (*r* = −0.223, *p* = 0.023), global beta1 (*r* = −0.226, *p* =0.022), and global beta2 (*r* = −0.250, *p* = 0.011). Among those beta bands, only middle beta2 was significantly correlated with affective lability (*r* = 0.212, *p* = 0.031) while other beta bands did not show any significant relationship with emotional problems. Affective lability was also significantly correlated with depression (*r* = 0.410, *p* < 0.001), state anxiety (*r* = 0.388, *p* <0.001), and trait anxiety (*r* =0.421, *p* < 0.001).

**Table 1:** M, SD, and correlation of key variables.

	CTQ	BDI	SAI	TAI	ALS	LF power	Middle beta2
CTQ	1						
BDI	0.470**	1					
SAI	0.349**	0.610**	1				
TAI	0.471**	0.710**	0.837**	1			
ALS	0.301**	0.410**	0.388**	0.421**	1		
LF Power	−0.227*	0.039	−0.017	−0.039	0.003	1	
Middle beta2	0.113	0.124	0.115	0.074	0.212*	−0.232*	1
M	42.29	7.73	36.25	39.35	16.37	502.65	3.86
SD	11.32	5.93	7.67	9.47	9.97	378.18	1.91

*p < 0.05.

**p < 0.001.

*CTQ, Childhood Trauma Questionnaire; BDI, Beck Depression Inventory; SAI, State Anxiety Inventory; TAI, Trait Anxiety Inventory; ALS, Affective Lability Scale; LF, low frequency (Hz); middle beta 2 (μV); M, mean*.

**Figure 2:**
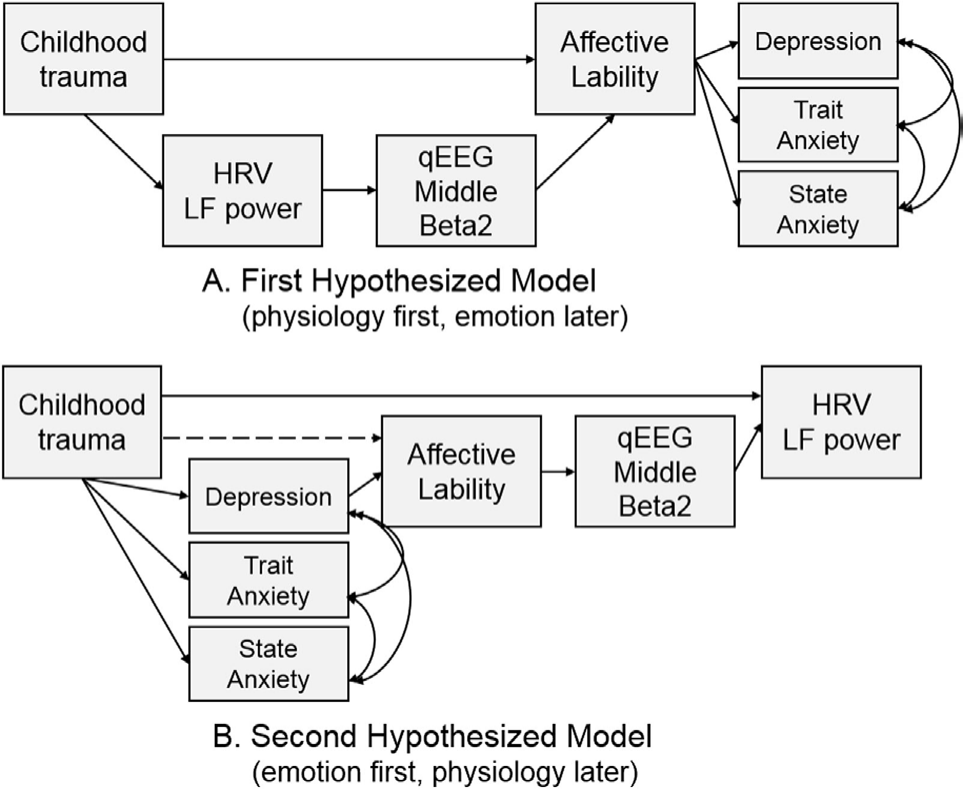
Two hypothesized models for path analysis.

Two models were hypothesized for the path analysis based on the correlation analyses. The first hypothesized model was composed of pathways from CTQ score to affective lability, from affective lability to middle beta2 activity, and from middle beta2 activity to LF power. The second hypothesized model was composed of pathways from CTQ score to LF power, from LF power to middle beta2 activity, and from middle beta2 activity to affective lability. Figure 2 shows two hypothesized models.

### Path Analysis

Hypothesized models were examined using path analysis. The first hypothesized model in Figure 2A was tested, and all pathways in the model were significant. The fitness indices of the model were as follows: χ^2^ = 29.403, df = 11, *p* = 0.002; CFI = 0.927; TLI = 0.860; RMSEA = 0.128 (confidence interval = 0.073–0.185); SRMR = 0.108; and AIC = 63.403. None of the fit indices me the fitness criteria.

The second hypothesized model (Figure 2B) was examined to compare it with the first model. The direct path from CTQ score to affective lability was not significant (*B* = 0.122, *p* = 0.172) and was excluded from the model, while all other pathways were significant. It showed great fit: χ^2^ = 12, *df* = 11, *p* = 0.363; CFI = 0.996; TLI = 0.992; RMSEA = 0.030 (confidence interval = 0.000–0.111); SRMR = 0.059; and AIC = 46.003. This second hypothesized model fit the data better than the first model since all fit indices met the criteria, and its AIC was much lower than that of the first model. Thus, the second hypothesized model was accepted.

**Figure 3:**
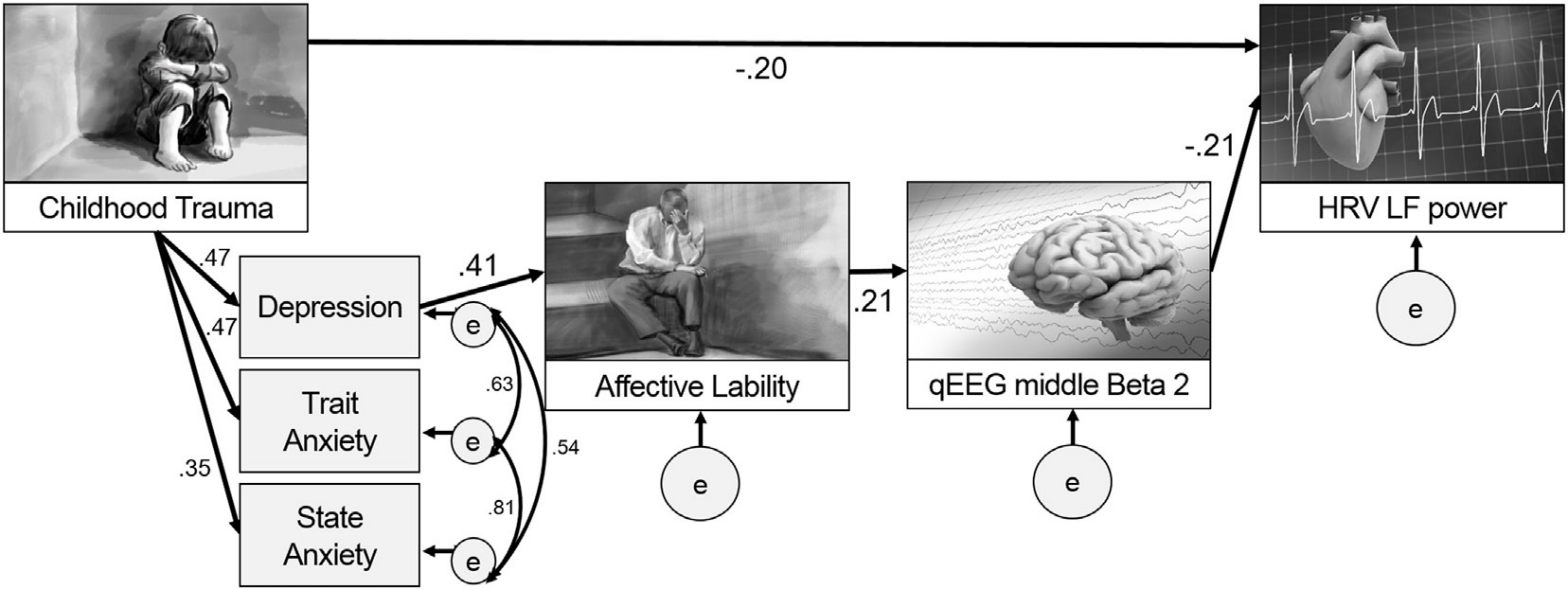
A comprehensive model from childhood trauma to heart rate variability (HRV) low frequency (LF) power with mediators including emotional problems and qEEG beta2 power.

Figure 3 presents the final model with the significant paths and standardized parameter estimates. There was a direct pathway from childhood trauma to LF power as well as an indirect pathway through the relation from depression and anxiety to affective lability, and from affective lability to middle beta2 activity. The standardized total effect of CTQ on LF power was −0.212, which consisted of −0.204 for the standardized direct effect and −0.009 for the standardized indirect effect. Table 2 shows the regression weights, SEs, and consistency ratios of the final model.

**Table 2:** Regression weight, standard error, and consistency ratio of the final model.

	Unstandardized regression weights	Standardized regression weights	SE	CR	*p*
CTQ → BDI	0.246	0.470	0.046	5.373	<0.001
CTQ → TAI	0.394	0.471	0.073	5.386	<0.001
CTQ → SAI	0.237	0.349	0.063	3.765	<0.001
BDI → ALS	0.690	0.410	0.152	4.544	<0.001
ALS → Middle beta2	0.041	0.212	0.019	2.192	0.028
Middle beta2 → LF power	−41.305	−0.210	18.643	−2.216	0.027
CTQ → LF power	−6.790	−0.204	3.151	−2.155	0.031
BDI error ↔ TAI error	27.174	0.627	5.063	5.367	<0.001
TAI error ↔ SAI error	20.087	0.539	4.191	4.792	<0.001
SAI error ↔ BDI error	48.439	0.814	7.598	6.375	<0.001

*CTQ, Childhood Trauma Questionnaire; BDI, Beck Depression Inventory; TAI, Trait Anxiety Inventory; SAI, State Anxiety Inventory; ALS, Affective Lability Scale; LF, low frequency*.

## Discussion

This study aimed to seek evidence for a model to explain paths after childhood trauma, including emotional problems such as affective lability, anxiety, and depression, and also physiological changes such as HRV and EEG in adulthood. Our major findings are as follows: (1) a model was developed including the paths from childhood trauma to HRV LF power in adulthood both directly and indirectly through depression, affective lability, and middle beta2 activity, which implies that child trauma could affect emotion first and then physiology; (2) specific pathways were clarified such that affective lability after childhood trauma and depression had a positive relationship with middle beta2 activity, and middle beta2 activity had a negative relationship with LF power; (3) the relationship between the middle beta2 of EEG and LF power of HRV was revealed, which could provide an insightful evidence supporting both the heart–brain connection and its relation to emotional problems such as affective lability.

The most interesting and important finding of this study is that it presented a path model for what can happen in adulthood emotionally and physiologically after childhood trauma. This study examined and compared two models. The first included paths from childhood trauma to emotional problems in adulthood with the mediating effect of heart and brain, and the second included paths from childhood trauma to heart changes with the mediating effect of emotion and the brain. Although both models were based on previous research (34, 35, 41), this study accepted the second model as significantly better after model comparison.

While previous studies have focused on whether childhood trauma is related to emotional problems, heart, and brain variables, respectively, this study proposed an extensive path model from childhood trauma to heart (LF power) with brain (beta2 power) as a mediators, which reflected the heart–brain connection. In addition to the direct relation between childhood trauma and HRV LF power later in adulthood, our results suggest that functional alterations of emotions, the brain, and the heart, in sequence, could also show progressive alterations pathophysiologically. Affective lability induced by childhood trauma (2, 5, 55) is known to be positively correlated with cortisol level (56), which is known to be associated with changes in EEG (57) and HRV indices (58). Although this study did not include cortisol level as a variable, relations with cortisol might explain this model and the relations among variables included in the final model.

The results of this study showed a negative relationship between childhood trauma and LF power. Previous studies have presented HRV as a stress indicator (58). In addition, previous research found that PTSD patients showed lower LF power (59) and adverse childhood experiences negatively influenced sympathetic cardiovascular activity (24). Since only LF, and not HF nor VLF, could reflect sympathetic cardiac influence (60), our result can be said to be in line with previous findings. It is suggested that LF could reflect a complex mix of sympathetic, parasympathetic, and other unidentified factors (20). LF is also proposed to reflect cardiac autonomic outflows by baroreflexes (61) and vagally mediated transmission between the heart and the central nervous system (62). Since only LF, and not HF or VLF, can be considered to represent body function more broadly, it would be more sensitive to changes after childhood trauma.

In our results, childhood trauma was related to depression, which was related to affective lability, and affective lability was related to middle beta2 activity. The lability of mood was found to be induced by childhood trauma (2, 5, 55). In addition to childhood trauma, EEG beta2 activity is known to be correlated with negative moods (12, 63, 64). Increased beta1 and beta2 activity was found in people with childhood maltreatment than in control group in both non-rapid eye movement sleep and in rapid eye movement sleep (65), and higher beta coherence was found in people with childhood trauma than those with adulthood traumas over right temporal–parietal areas, and those with no trauma history over the right temporal region (66). Childhood trauma can induce various responses, including physiological hyperarousal and dissociation (67), and hyperarousal is related to depression and anxiety (68). Since an increase in beta activity is also likely to be an index of central nervous system hyperarousal in people with a history of trauma (69), the results of this study showing relations among childhood trauma, emotional problems, and beta activity could be explained by hyperarousal.

In this study, the middle beta2 frequency showed a significant negative relation with the LF power of HRV. While previous studies have only examined the simple correlation between childhood trauma and heart and brain functions, respectively (70), our path analysis suggest a mediating sequence whereby childhood trauma might influence the brain through emotions, which in turn could affects the heart. This is in line with previous research in the coherence of emotion recognition, brain, and the heart (40). A notable finding is that only the beta2 band was involved in this path model. Considering that beta2 reflects alertness and anxious states (71) resulting from changes in the autonomic nervous system related to LF power, this result is quite reliable. In addition, only the middle part of the brain surface showed significant results. Excessive beta is usually observed in central sites as well as frontal sites in patients with anxiety disorders (72). One study examined increased beta2 activity at the C4 and F4 electrodes in patients with major depression (73). This evidence suggests that the middle central electrode area reflects emotional problems as well as HRV changes.

This study has some limitations. First, the subjects were non-clinical volunteers with different levels of childhood trauma severity. Although the CTQ scores were normative and statistically appropriate in this study, they could not represent aspects of clinical samples, including PTSD patients. We expect to find other interesting findings, including the investigation of the relations between subscales of CTQ and physiological parameters, in further studies with PTSD patients. Moreover, childhood trauma was assessed retrospectively. Despite the fact that CTQ is a well-validated and stable measurement over the time (74), a longitudinal study with a prospective design could address these issues more appropriately. In addition, EEG and HRV data were not collected simultaneously. Since HRV was measured right after EEG recording, participants did not seem to show any change in their state. Accordingly, further studies should control this process more thoroughly. Lastly, our study could not explore regional brain alterations in participants with childhood trauma. Source localization methods would be helpful for understanding regional brain changes in these participants.

In conclusion, to our knowledge, this is the first study to elucidate a path model from childhood trauma to adult HRV LF power with mediating factors, such as emotional problems and EEG beta2 activity. The results of this study suggests that trauma history in childhood could induce emotional problems, including depression, anxiety, and affective lability, and affective lability could be related to the middle beta2 in EEG, which could later be associated with LF power in adulthood. Our results provide insight into the heart–brain connection and its relation with childhood trauma and emotion, which could be a steppingstone for further research.

## Ethics Statement

This study was carried out in accordance with the recommendations of Institutional Review Board of Inje University with written informed consent from all subjects. All subjects gave written informed consent in accordance with the Declaration of Helsinki. The protocol was approved by the Institutional Review Board (2015-07-026-001).

## Author Contributions

MJ suggested the idea, conducted the experiment, analyzed the results, and wrote the whole manuscript. JK and MH edited the manuscript. SK analyzed the HRV and qEEG data. S-WH designed the study and edited the manuscript.

## Conflict of Interest Statement

The authors declare that the research was conducted in the absence of any commercial or financial relationships that could be construed as a potential conflict of interest.
