# Supplementary material for: An Integrated Model of Emotional Problems, Beta Power of Electroencephalography, and Low Frequency of Heart Rate Variability after Childhood Trauma in a Non-Clinical Sample: A Path Analysis Study
Source: Front Psychiatry. 2018 Jan 22;8:314. doi: 10.3389/fpsyt.2017.00314 (PMC5786859; doi:10.3389/fpsyt.2017.00314)
Supplement: Supplementary file 1 [file table_1.docx]

Supplementary Table.

*Correlation coefficients of all variables.*

|  | **1. CTQ** | **2. BDI** | **3. SAI** | **4. TAI** | **5. ALS** | **6. VLF power** | **7 LF power** | **8 HF power** | **9. anterior delta** | **10. anterior theta** | **11. anterior low alpha** | **12. anterior high alpha** | **13. anterior beta1** | **14.anterior beta2** | **15. anterior beta3** | **16. anterior gamma** | **17. middle delta** | **18. middle theta** | **19. middle low alpha** | **20. middle high alpha** | **21. middle beta1** | **22. middle beta2** | **23. middle beta3** | **24. middle gamma** | **25. posterior delta** | **26. posterior theta** | **27. posterior low alpha** | **28. Posterior high alpha** | **29. posterior beta1** | **30. posterior beta2** | **31. posterior beta3** | **32. posterior gamma** | **33. global delta** | **34. global theta** | **35. global low alpha** | **36. global high alpha** | **37. global beta1** | **38. global beta2** | **39. global beta3** | **40. global gamma** |
| --- | --- | --- | --- | --- | --- | --- | --- | --- | --- | --- | --- | --- | --- | --- | --- | --- | --- | --- | --- | --- | --- | --- | --- | --- | --- | --- | --- | --- | --- | --- | --- | --- | --- | --- | --- | --- | --- | --- | --- | --- |
| **1** | 1 |  |  |  |  |  |  |  |  |  |  |  |  |  |  |  |  |  |  |  |  |  |  |  |  |  |  |  |  |  |  |  |  |  |  |  |  |  |  |  |
| **2** | 478** | 1 |  |  |  |  |  |  |  |  |  |  |  |  |  |  |  |  |  |  |  |  |  |  |  |  |  |  |  |  |  |  |  |  |  |  |  |  |  |  |
| **3** | .340** | .598** | 1 |  |  |  |  |  |  |  |  |  |  |  |  |  |  |  |  |  |  |  |  |  |  |  |  |  |  |  |  |  |  |  |  |  |  |  |  |  |
| **4** | .477** | .713** | .828** | 1 |  |  |  |  |  |  |  |  |  |  |  |  |  |  |  |  |  |  |  |  |  |  |  |  |  |  |  |  |  |  |  |  |  |  |  |  |
| **5** | .312** | .420** | .378** | .428** | 1 |  |  |  |  |  |  |  |  |  |  |  |  |  |  |  |  |  |  |  |  |  |  |  |  |  |  |  |  |  |  |  |  |  |  |  |
| **6** | -.124 | .114 | .112 | .103 | .127 | 1 |  |  |  |  |  |  |  |  |  |  |  |  |  |  |  |  |  |  |  |  |  |  |  |  |  |  |  |  |  |  |  |  |  |  |
| **7** | -.222* | .042 | -.018 | -.037 | .006 | .395** | 1 |  |  |  |  |  |  |  |  |  |  |  |  |  |  |  |  |  |  |  |  |  |  |  |  |  |  |  |  |  |  |  |  |  |
| **8** | -.061 | .104 | -.128 | -.121 | .162 | .421** | .408** | 1 |  |  |  |  |  |  |  |  |  |  |  |  |  |  |  |  |  |  |  |  |  |  |  |  |  |  |  |  |  |  |  |  |
| **9** | -.005 | -.133 | -.090 | -.015 | -.099 | -.100 | -.185 | -.160 | 1 |  |  |  |  |  |  |  |  |  |  |  |  |  |  |  |  |  |  |  |  |  |  |  |  |  |  |  |  |  |  |  |
| **10** | .027 | .025 | .032 | .084 | -.196* | -.158 | -.096 | -.147 | .445** | 1 |  |  |  |  |  |  |  |  |  |  |  |  |  |  |  |  |  |  |  |  |  |  |  |  |  |  |  |  |  |  |
| **11** | .014 | -.046 | .030 | -.045 | .051 | .109 | .144 | .104 | -.738** | -.364** | 1 |  |  |  |  |  |  |  |  |  |  |  |  |  |  |  |  |  |  |  |  |  |  |  |  |  |  |  |  |  |
| **12** | -.049 | .153 | -.005 | .010 | .101 | .044 | .099 | .132 | -.246* | -.437** | -.381** | 1 |  |  |  |  |  |  |  |  |  |  |  |  |  |  |  |  |  |  |  |  |  |  |  |  |  |  |  |  |
| **13** | .072 | .056 | .074 | .090 | -.089 | -.141 | -.230* | -.162 | .478** | .308** | -.630** | .036 | 1 |  |  |  |  |  |  |  |  |  |  |  |  |  |  |  |  |  |  |  |  |  |  |  |  |  |  |  |
| **14** | .051 | .081 | .096 | .063 | .064 | -.019 | -.260** | -.110 | .332** | .119 | -.458** | -.001 | .671** | 1 |  |  |  |  |  |  |  |  |  |  |  |  |  |  |  |  |  |  |  |  |  |  |  |  |  |  |
| **15** | .050 | .070 | .081 | .092 | .050 | -.118 | -.201* | -.229* | .453** | .272** | -.555** | -.046 | .604** | .666** | 1 |  |  |  |  |  |  |  |  |  |  |  |  |  |  |  |  |  |  |  |  |  |  |  |  |  |
| **16** | .019 | .137 | .068 | .091 | .097 | -.114 | -.151 | -.168 | .412** | .219* | -.455** | -.089 | .494** | .509** | .844** | 1 |  |  |  |  |  |  |  |  |  |  |  |  |  |  |  |  |  |  |  |  |  |  |  |  |
| **17** | -.061 | -.174 | -.162 | -.077 | -.156 | -.116 | -.154 | -.132 | .927** | .503** | -.694** | -.248* | .434** | .242* | .439** | .377** | 1 |  |  |  |  |  |  |  |  |  |  |  |  |  |  |  |  |  |  |  |  |  |  |  |
| **18** | .005 | -.031 | .018 | .053 | -.265** | -.163 | -.081 | -.160 | .454** | .940** | -.325** | -.465** | .277** | .089 | .249* | .196* | .538** | 1 |  |  |  |  |  |  |  |  |  |  |  |  |  |  |  |  |  |  |  |  |  |  |
| **19** | .007 | -.050 | .048 | -.043 | .020 | .103 | .122 | .086 | -.689** | -.315** | .973** | -.416** | -.612** | -.435** | -.556** | -.468** | -.684** | -.286** | 1 |  |  |  |  |  |  |  |  |  |  |  |  |  |  |  |  |  |  |  |  |  |
| **20** | -.006 | .194* | .037 | .077 | .152 | .089 | .129 | .127 | -.275** | -.448** | -.314** | .941** | .006 | -.002 | -.064 | -.115 | -.324** | -.527** | -.367** | 1 |  |  |  |  |  |  |  |  |  |  |  |  |  |  |  |  |  |  |  |  |
| **21** | .103 | .060 | .027 | .060 | .025 | -.183 | -.243* | -.121 | .447** | .132 | -.625** | .185 | .849** | .532** | .518** | .457** | .356** | .107 | -.648** | .134 | 1 |  |  |  |  |  |  |  |  |  |  |  |  |  |  |  |  |  |  |  |
| **22** | .088 | .099 | .122 | .056 | .184 | -.029 | -.233* | -.084 | .252** | -.054 | -.426** | .160 | .543** | .837** | .503** | .487** | .11 | -.078 | -.430** | .105 | .616** | 1 |  |  |  |  |  |  |  |  |  |  |  |  |  |  |  |  |  |  |
| **23** | .087 | .093 | .062 | .067 | .146 | -.112 | -.222* | -.146 | .417** | .140 | -.543** | .054 | .550** | .643** | .867** | .845** | .343** | .096 | -.564** | .002 | .624** | .714** | 1 |  |  |  |  |  |  |  |  |  |  |  |  |  |  |  |  |  |
| **24** | .061 | .103 | .038 | .045 | .191 | -.097 | -.169 | -.063 | .328** | .043 | -.400** | .057 | .377** | .404** | .571** | .783** | .228* | -.002 | -.434** | -.014 | .562** | .664** | .851** | 1 |  |  |  |  |  |  |  |  |  |  |  |  |  |  |  |  |
| **25** | -.073 | -.205* | -.162 | -.132 | -.186 | -.151 | -.122 | -.170 | .863** | .510** | -.639** | -.268** | .440** | .237* | .431** | .365** | .923** | .548** | -.625** | -.347** | .361** | .133 | .341** | .243* | 1 |  |  |  |  |  |  |  |  |  |  |  |  |  |  |  |
| **26** | .007 | -.088 | -.067 | -.030 | -.263** | -.189 | -.032 | -.172 | .474** | .799** | -.269** | -.500** | .254** | .096 | .250* | .200* | .537** | .885** | -.238* | -.556** | .111 | -.052 | .103 | .034 | .652** | 1 |  |  |  |  |  |  |  |  |  |  |  |  |  |  |
| **27** | -.006 | -.057 | .049 | -.050 | -.015 | .077 | .040 | .063 | -.605** | -.223* | .916** | -.469** | -.544** | -.416** | -.522** | -.429** | -.582** | -.193* | .941** | -.437** | -.590** | -.421** | -.523** | -.388** | -.539** | -.172 | 1 |  |  |  |  |  |  |  |  |  |  |  |  |  |
| **28** | .014 | .198* | .057 | .131 | .196* | .092 | .107 | .162 | -.189 | -.356** | -.342** | .846** | .015 | .053 | -.039 | -.08 | -.230* | -.436** | -.384** | .886** | .135 | .151 | .027 | .004 | -.361** | -.577** | -.509** | 1 |  |  |  |  |  |  |  |  |  |  |  |  |
| **29** | .119 | -.009 | -.002 | .014 | -.055 | -.120 | -.177 | -.156 | .500** | .150 | -.607** | .129 | .716** | .449** | .519** | .390** | .421** | .142 | -.615** | .094 | .832** | .463** | .558** | .390** | .448** | .188 | -.619** | .039 | 1 |  |  |  |  |  |  |  |  |  |  |  |
| **30** | .063 | .025 | .049 | .016 | .034 | -.008 | -.180 | -.151 | .406** | .065 | -.365** | -.113 | .483** | .707** | .544** | .512** | .279** | .084 | -.355** | -.134 | .477** | .742** | .641** | .526** | .292** | .135 | -.392** | -.146 | .547** | 1 |  |  |  |  |  |  |  |  |  |  |
| **31** | .056 | .054 | .010 | .048 | .067 | -.106 | -.165 | -.197* | .590** | .313** | -.595** | -.095 | .592** | .578** | .759** | .731** | .525** | .289** | -.589** | -.141 | .603** | .566** | .807** | .677** | .546** | .339** | -.593** | -.148 | .671** | .774** | 1 |  |  |  |  |  |  |  |  |  |
| **32** | -.013 | .052 | -.015 | .021 | .043 | -.114 | -.098 | -.160 | .521** | .304** | -.466** | -.164 | .470** | .413** | .635** | .715** | .479** | .288** | -.465** | -.224* | .489** | .449** | .711** | .702** | .511** | .355** | -.467** | -.231* | .510** | .658** | .939** | 1 |  |  |  |  |  |  |  |  |
| **33** | -.049 | -.179 | -.146 | -.079 | -.148 | -.130 | -.161 | -.157 | .956** | .503** | -.712** | -.260** | .468** | .274** | .456** | .401** | .981** | .531** | -.689** | -.328** | .408** | .172 | .385** | .289** | .963** | .577** | -.594** | -.275** | .476** | .335** | .575** | .527** | 1 |  |  |  |  |  |  |  |
| **34** | .012 | -.038 | -.008 | .035 | -.249* | -.181 | -.077 | -.167 | .494** | .947** | -.347** | -.485** | .303** | .116 | .279** | .228* | .563** | .980** | -.306** | -.534** | .141 | -.046 | .135 | .047 | .611** | .942** | -.218* | -.480** | .184 | .112 | .343** | .345** | .577** | 1 |  |  |  |  |  |  |
| **35** | .009 | -.048 | .049 | -.044 | .021 | .095 | .095 | .086 | -.683** | -.306** | .978** | -.434** | -.602** | -.437** | -.553** | -.456** | -.662** | -.273** | .988** | -.385** | -.628** | -.424** | -.548** | -.407** | -.609** | -.231* | .975** | -.427** | -.627** | -.375** | -.602** | -.474** | -.673** | -.296** | 1 |  |  |  |  |  |
| **36** | -.008 | .196* | .039 | .091 | .172 | .089 | .122 | .152 | -.241* | -.419** | -.354** | .947** | .017 | .026 | -.051 | -.096 | -.276** | -.489** | -.400** | .969** | .153 | .151 | .03 | .018 | -.350** | -.574** | -.495** | .965** | .079 | -.139 | -.136 | -.218* | -.302** | -.517** | -.430** | 1 |  |  |  |  |
| **37** | .102 | .018 | .019 | .040 | -.059 | -.155 | -.225* | -.172 | .507** | .195* | -.657** | .136 | .875** | .548** | .567** | .448** | .433** | .181 | -.663** | .095 | .937** | .538** | .591** | .432** | .446** | .195* | -.631** | .067 | .954** | .533** | .662** | .511** | .482** | .215* | -.662** | .095 | 1 |  |  |  |
| **38** | .068 | .056 | .076 | .031 | .058 | -.028 | -.249* | -.143 | .383** | .074 | -.441** | -.033 | .604** | .905** | .624** | .540** | .254** | .071 | -.424** | -.056 | .559** | .890** | .702** | .535** | .260** | .096 | -.429** | -.03 | .530** | .928** | .715** | .565** | .306** | .097 | -.433** | -.038 | .580** | 1 |  |  |
| **39** | .053 | .031 | .016 | .038 | .029 | -.117 | -.183 | -.231* | .494** | .252** | -.521** | -.100 | .551** | .572** | .902** | .853** | .449** | .232* | -.525** | -.133 | .530** | .526** | .898** | .708** | .443** | .251* | -.511** | -.144 | .588** | .724** | .899** | .815** | .482** | .269** | -.529** | -.136 | .588** | .684** | 1 |  |
| **40** | .005 | .096 | .037 | .060 | .073 | -.145 | -.173 | -.171 | .562** | .310** | -.565** | .102 | .540** | .522** | .747** | .853** | .510** | .286** | -.568** | -.166 | .558** | .567** | .832** | .812** | .516** | 309** | -.538** | -.132 | .508** | 648** | .908** | .933** | .553** | .331** | -.563** | .138 | .546** | .630** | .860** | 1 |

* *P* < 0.05, ** *P* < 0.001. CTQ, Childhood Trauma Questionnaire; BDI, Beck Depression Inventory; SAI, State Anxiety Inventory; TAI, Trait Anxiety Inventory; ALS, Affective Lability Scale; VLF, very low frequency (Hz); LF, low frequency (Hz); HF, high frequency (Hz); electroencephalogram (μV)
